# Spiritual counselling and women's well-being in orthodox christianity: embryo adoption as surrogate motherhood

**DOI:** 10.3389/fgwh.2026.1863643

**Published:** 2026-06-03

**Authors:** Roman Tarabrin

**Affiliations:** Institute of Social Sciences, Sechenov First Moscow State Medical University, Moscow, Russia

**Keywords:** embryo adoption, orthodox christianity, spiritual counselling, surrogate motherhood, women's well-being

## Abstract

This article examines the role of spiritual counselling in addressing infertility-related suffering among Orthodox Christian women, with particular attention to surrogacy and embryo adoption. Infertility may generate not only psychological distress but also profound moral and religious conflict when assisted reproductive technologies are perceived as incompatible with faith commitments. Focusing on Orthodox Christianity, the article argues that counselling must integrate theological, ethical, and clinical dimensions rather than reducing religious concerns to psychological support alone. Using a case-based approach, it shows how pastoral counselling can help infertile couples navigate doctrinal restrictions, especially where local Orthodox positions on assisted reproduction are diverse or unclear. Using a specific clinical case, the authors demonstrate how spiritual guidance enabled a couple to consider embryo adoption, a solution that is technically equivalent to surrogacy, as permissible within their religious framework. The article concludes that spiritually informed counselling can reduce distress, support informed decision-making, and improve the well-being of infertile women while remaining faithful to Orthodox moral teaching.

## Introduction

Infertility is a condition that can result in significant psychological distress for married couples. This distress can manifest as anxiety, stress, and depression (1). The intense transcendent desire to become a parent is revealed in the form of “loss of purpose, loss of normal reality, and the loss of the experience that time flows toward a meaningful future” (2). The integration of a religious dimension into this framework serves to exacerbate the psychological suffering experienced by an individual, thereby compounding the moral suffering. While psychological suffering may be alleviated through professional support from a psychologist or psychotherapist, the same cannot be said for moral suffering. In such cases, the only effective remedy may be to address the underlying cause by turning to religion.

However, in instances where religious denomination prohibits the use of reproductive technologies, a childless couple faces a moral dilemma: to have a child despite the religious restrictions on them, or to remain childless in accordance with their religious tradition. For devoutly religious individuals, the transgression of religious principles and the decision to seek medical intervention for the purpose of procreation would likely engender profound moral distress in the future.

The same is true for medical professionals. Religious issues cannot be ignored in treatment plans since they directly affect psychological suffering, negatively influencing the chances of conception (3). In the research literature, practical counselling implications are mentioned, such as the importance of counselors discussing faith-specific standards to align treatment with spiritual beliefs, fostering trust and adherence in the patient. This also prevents distress and legal issues in religious communities and enhances holistic care in multicultural settings (4). It has been demonstrated that stronger religious observance and religious beliefs positively correlate with problem-solving coping. Similarly, positive religious coping (e.g., seeking spiritual support, benevolent religious reappraisal) is associated with better adjustment outcomes, including lower psychological distress and more favorable emotional functioning (5). On the other hand, multiple papers conclude that religious/spiritual issues are systematically under-recognized in infertility practice and that this gap has important implications for patient well-being and professional guidelines (6–9).

Such considerations highlight the practical necessity for clinicians to include spiritual counselling. This is particularly applicable in diverse societies. For example, Shenker argues that in multicultural societies such as Israel, where Jews, Muslims, and Christians coexist, physicians encounter a variety of beliefs. To avoid conflict and maintain long-term consensus between patients and physicians on treatment, an understanding of religious issues is essential (10). Contemporary nations in general, with several exceptions, represent multi-ethnic, multi-cultural societies. This is why navigation of religious issues influencing patients' adherence to fertility treatment is crucial. Infertility counselling must probe religious views early, as unaddressed conflicts lead to denial of care, ethical dilemmas, or suboptimal outcomes like embryo wastage refusals.

## Methods

The paper uses a case-based qualitative approach centered on a clinical counseling case, supported by theological and philosophical analysis of how spiritually informed counseling can guide decision-making about embryo adoption/surrogacy within Orthodox moral constraints.

In this study, we have selected Orthodox Christianity as a case in point. Orthodox Christianity is a traditional Christian denomination with a conservative position on the use of reproductive technologies. Some variants of ART are permitted, but most are not. Orthodox believers (260 million people worldwide, their number having doubled in the last century) (11) live in various cultures around the world. However, despite their diverse contexts, Orthodox Christians share common characteristics. These include relying on holy scriptures (New and Old Testaments) and holy tradition for guidance. The holy tradition includes canonical texts and rules of ecumenical councils, writings of the holy fathers of the first and second millennia, prayers, and liturgical texts (12, 13). The final normative role for newly emerging issues belongs to the council of all Orthodox bishops, as the expression of the Holy Spirit's presence in the Church and the action of God among people who are seeking to attain holiness. While different sociocultural issues affect the form of Orthodox communities around the world to a slight degree, the aforementioned peculiarities enable them to be analyzed as a single religious community. The largest numbers of Orthodox Christians live in Russia, Ethiopia, Ukraine, Romania, Greece, Serbia, Bulgaria, and Belarus, according to Pew Research Center reports (11). Since Russia is home to the largest number of adherents to this religion, we have chosen this country as an illustrative example to demonstrate that the results of our study will help promote women's well-being in other highly conservative countries where Orthodox women may suffer in the event of infertility. Additionally, our results could be applied to Western countries such as the US, where a growing interest in Orthodox Christianity is being noted and where the number of Orthodox believers are gradually increasing (14).

## Results

### Orthodox christianity and assisted reproductive technologies (ART)

In Orthodox Christianity, the use of *in vitro* fertilization (IVF) is permitted, provided that the number of embryos created is limited to those that will subsequently be transferred to the biological mother's uterus (12, 15). Surrogacy is considered unacceptable. In particular, the Russian Orthodox Church, the largest of all regional Orthodox churches, clearly forbids the use of a surrogate mother to carry a child (16). Among the reasons cited are 1) the interference of a third party in the marital relationship, which is equated with adultery; 2) the commodification of the mother and child; and 3) the destruction of the bond formed between the surrogate mother and the child (17).

### Orthodox pastoral counselling as a particular case of spiritual counselling

In a conservative religious context, a religious woman who is infertile may experience spiritual suffering, which should be addressed with a holistic approach. This may be the responsibility of a spiritual counsellor; however, it is essential that the reasoning behind the counselling be grounded in a coherent and substantiated framework. An Orthodox counsellor is unable to refer to church decrees, as such decrees are generally unavailable. There is no extant pan-Orthodox document that is both fully harmonized and universally accepted. Researchers have highlighted that, since the last ecumenical council comprising representative bishops from all regional Orthodox churches was convened over twelve hundred years ago, it is the responsibility of the regional churches to make their local statements on how to use ART (13). All Orthodox churches are based on the same religious principles. However, regional differences in society and culture mean that Orthodox churches in different regions issue their own texts which, while agreeing on the core principles, differ in detail. It is evident that, amongst all Orthodox churches, it is the Greek Orthodox Church alone that has articulated its position on IVF with relative clarity (18). The Russian Orthodox Church adopts a contradictory position, and the ongoing discourse on the ethical considerations surrounding IVF, initiated in 2021, has not yet culminated in the establishment of explicit guidelines for infertile religious couples (17). The Orthodox Church in America does not possess an official document; there are only outlined provisions regarding ART from one of the members of its medical commission (19). In a similar vein, the Serbian Orthodox Church has only recently begun to articulate a more nuanced set of views, albeit from a limited number of theologians (20). The Romanian Orthodox Church, in common with many other Orthodox Churches, has not adopted an official position on the matter (21). Moreover, the existing documents fail to address the plethora of ethical issues encountered in clinical practice. Consequently, a case-by-case approach is necessary during spiritual counselling.

However, spiritual counselling in Orthodoxy should be understood quite differently from that provided by chaplains in hospitals. First of all, Orthodox spiritual counselling is not the psychological reduction of spiritual foundations of bioethical issues. In Orthodoxy, a counsellor should address religious issues from the standpoint of having a pastor's position of authority (22). This is why counsellors are usually pastors, making them more accessible and trustable. This fact correlates with clergy as counsellors in many other Christian denominations (23).

Secondly, Orthodox spiritual counselling focuses on the treatment of a soul rather than the psychological consolation of a patient. Such treatment is provided in Church sacraments (such as confession and Holy Communion), and therefore Church mysteries become a major spiritual means provided by the “hospital” of the Orthodox Church to help an infertile couple overcome childlessness (13).

And finally, in Orthodoxy, spiritual counselling is based on the image of God which informs that counselling (22). Based on the Christian teaching of a human being as bearing the image of God, and taking into account other doctrines such as the purpose of marriage and the proper use of medicine, one can at least define the lines that should not be crossed in the use of assisted reproduction (24). It implies that in the absence of normative regulations in a local church, a counsellor may discern what is absolutely ethically unacceptable and what is permissible under some circumstances. In this regard, when providing spiritual counselling, it is possible to apply the concept of pastoral oikonomia (pastoral flexibility). This term refers to the adaptation of different rules for different people, depending on their spiritual abilities and other circumstances. When a medical procedure is not inherently forbidden, its use can still have different spiritual effects depending on a person's spiritual progress. For some, such a procedure might impede their progress toward God, while for others it could be beneficial (or at least not harmful) in their journey toward holiness. Thus, a counsellor might regard some practices permissible and others not depending on the situation (13).

Counselling may also facilitate the provision of options for religious infertile women who are unable to utilize standard ART methods to conceive. This enables the application of extant normative regulations on reproductive technologies in Orthodoxy, or the selection of treatment options that would be consistent with the woman's spiritual maturity. Sometimes, given opposing views within a local Orthodox church, a counsellor can help choose the reproductive option most suitable for that particular case.

In the subsequent section of this article, an example is employed of counselling an infertile couple who identified as Orthodox Christians but were unable to conceive using standard IVF procedures.

### Description of the case

The authors of this paper counseled E. and A. (a 28-year-old man and a 27-year-old woman) who had been married for five years prior to the consultation. They were Orthodox Christians for whom religion played a vital role in their life, shaping their spiritual well-being, sense of purpose, and overall fulfillment. They regularly participated in church rituals, so their psychological distress from infertility was compounded by religious suffering. Their deep desire to have a child triggered a profound internal moral crisis. They could not adopt a child because E. was the only son in his family, and their relatives were closely monitoring their conception process and potential pregnancy. According to them, adoption would only have been possible if they could have left their city for a year in order to keep the adoption a secret from their parents and relatives and then returned with their adopted child. However, this was impossible due to their work commitments.^1^

Although the Russian Orthodox Church lacks a clear official position on reproductive technologies, several studies have highlighted a consensus that, in Orthodoxy, the freezing of embryos is not permitted, which has led to difficulties with the use of IVF in a stimulated cycle (24). In light of this, during the initial stage of spiritual counselling, it was suggested that she undergo IVF in a natural cycle. Although clinics are very reluctant to agree to IVF in a natural cycle—since only one egg is produced, which reduces the chances of conception—we managed to find an experienced doctor who was willing to conduct the IVF procedure while taking into account the aforementioned Orthodox restrictions.

Two oocytes were retrieved from A. during the natural cycle; they were fertilized, resulting in two high-quality embryos. The transfer was performed on day 5. However, the resulting twin pregnancy stopped developing at 8 weeks, which caused severe psychological distress. The couple attempted IVF two more times in a natural cycle, categorically refusing the doctor's suggestions to obtain additional embryos due to religious considerations, as Orthodox Christianity holds that a human being comes into existence from the moment of conception.

During the second stage of spiritual counselling, we suggested transferring donor embryos. We were unsure whether the couple would agree to this, as they were adamant about having their own biological child. However, considering that they were also open to adopting a child but had not done so due to concerns that their relatives might learn and reveal the secret of the adoption, we proposed the option of transferring a donor embryo—a practice known in the United States as “Snowflake adoption” (25).

## Discussion

### Surrogate motherhood as embryo adoption in orthodoxy

The transfer of thawed embryos into the uterus of the adoptive mother, as well as pregnancy, childbirth, and raising of the child, will take place as mandatory steps in a surrogacy procedure. Although the process of becoming pregnant with a donor embryo does not technically differ from surrogacy, pregnancy through the adoption of a cryopreserved embryo is fundamentally different because of the purpose of the procedure according to Orthodox considerations (26). In surrogacy, the embryo is carried to term for transfer to adoptive parents. In contrast, in snowflake adoption, pregnancy with the embryo occurs after its adoption and represents a last-ditch effort to give the embryo a chance to develop. Consequently, it constitutes an attempt to save a human life.

Nevertheless, a woman who adopts an embryo (much like a surrogate mother) carries another person's embryo in her womb and thus participates in the reproductive process of the married couple who conceived the embryo; thereby, according to Orthodox teaching, she defiles their marital bed. For this reason, the adoption of cryopreserved embryos is opposed by contemporary Orthodox bioethicists. Thus, Prof. H. T. Engelhardt writes: “Surrogate motherhood, even if undertaken in order to rescue an unborn child, would still involve a significant dissociation of reproduction from the unity of marriage. Though the acceptance of zygotes for surrogate gestation could be interpreted as the saving of early human life, one must avoid immoral actions, even if these will save the life of others” (12).

It is worth noting that Engelhardt's argument regarding the involvement of a third-party woman in another person's reproductive process is tempered by his own analysis of the case of the biological mother's sudden death. In such a scenario, the life of an already conceived embryo may be preserved by having another woman carry it to term. Engelhardt confirms the ethical acceptability of this scenario by likening it to a nanny breastfeeding a newborn when the mother is unable to do so. [(12), p. 257].

Any attempt to determine the ethical acceptability or unacceptability of surrogacy in addressing the issue of frozen embryos requires, first, a decision on which is the lesser evil: the death of a human being or an intrusion into a marital relationship—and second, what are the intentions behind third-party intervention in the couple's relationship? We sought answers to these questions in academic discussions within Orthodox bioethics. In another article, an author argues that embryo adoption is permissible in Orthodoxy because 1) saving the life of a human being, the bearer of God's image, is far more valuable than interfering in the reproductive process; and 2) the intrusion into the marital relationship occurs out of necessity, because the parents have died or are no longer able or willing to carry a child to term (26). During the counselling process, the couple agreed that in such a case, the issue at hand is the salvation of a child frozen at the embryonic stage. At the same time, the intrusion into the reproductive process is unintentional.

However, since Russian law does not provide for adoption at the embryonic stage, frozen embryos are treated as “property,” i.e., as donor material. Consequently, all legal regulations governing tissue donation apply to donor embryos, namely, the age of the donors (i.e., the biological father and mother) must be under 35, and an embryo must have been tested for chromosomal abnormalities to prevent the conception of a child with a severe congenital disorder. These two conditions together made the practical implementation of our proposal unlikely, since people over 35 typically enter IVF programs, and therefore frozen embryos that are rejected by biological parents immediately fall outside the scope of legal authorization for transfer. Genetic testing is an expensive procedure, so it is typically performed for parents over 40 or in cases of strict medical indications, such as being a carrier of a specific gene. Thus, finding a tested embryo whose parents were younger than 35 was extremely difficult. Moreover, the couple seeking counselling was categorically opposed to prenatal genetic testing, as they viewed this procedure as a form of embryo selection for religious reasons. However, they were willing to have an embryo that had been tested without their consent transferred.

Follow-up. An embryo that complied with the legal regulations governing donation was found at one of the clinics and transferred to A.'s uterus. A baby girl was born via natural childbirth. At the time of writing, the child is over 2 years old. The parents are happy.

## Conclusion

Preserving a woman's religious identity and respecting her spiritual beliefs can alleviate emotional distress and increase the likelihood of her acceptance of fertility treatment. In this context, spiritual counselling serves as a vital tool for assisting infertile women within Orthodox Christianity, as it helps reconcile medical options with religious and cultural values. In the Orthodox context, spiritual care must be grounded in a theological understanding of the human person, marriage, and the permissible limits of medical intervention, rather than being reduced to mere psychological support.

The clinical case discussed here demonstrates that, in the absence of a unified and clear position from Orthodox churches, individual counselling is possible to help find a solution compatible with the couple's faith and conscience. The case of spiritual counselling discussed here demonstrates that even within a religion as conservative as Orthodox Christianity, the use of surrogacy is possible, provided that the child born is raised by the birth mother as a full-fledged child.

## Data Availability

The original contributions presented in the study are included in the article/Supplementary Material, further inquiries can be directed to the corresponding author/s.

## Footnotes

^1^It is important to note that the opposition to adoption described here is an idiosyncratic fact affecting only these patients' families. In contemporary Russia, there is a greater level of acceptance of adoption. For this reason, we typically initiate our consultation with a discussion of the adoption of a child who has already been born. However, it has also been observed that individuals seeking spiritual counsel have typically ruled out adoption, either due to a strong desire to have biological offspring or an inability to care for a stranger's child.
